# Uptake of meta-iodobenzylguanidine in neuroendocrine tumours is mediated by vesicular monoamine transporters

**DOI:** 10.1038/sj.bjc.6601276

**Published:** 2003-09-30

**Authors:** L Kölby, P Bernhardt, A-M Levin-Jakobsen, V Johanson, B Wängberg, H Ahlman, E Forssell-Aronsson, O Nilsson

**Affiliations:** 1 Department of Surgery, Göteborg University, Sahlgrenska University Hospital, SE-413 45 Göteborg, Sweden; 2 Department of Radiation Physics, Pathology Lundberg Laboratory for Cancer Research, Göteborg University, Sahlgrenska University Hospital, SE-413 45 Göteborg, Sweden; 3 Department of Pathology Lundberg Laboratory for Cancer Research, Göteborg University, Sahlgrenska University Hospital, SE-413 45 Göteborg, Sweden

**Keywords:** meta-iodobenzylguanidine, MIBG, nude mice, carcinoid tumours, pheochromocytoma, vesicular monoamine transporter, VMAT

## Abstract

The radio-iodinated noradrenaline analogue meta-iodobenzylguanidine (MIBG) can be used for scintigraphy and radiation therapy of neuroendocrine (NE). The aim of the present study was to study the importance of vesicular monoamine transporters (VMATs) for the uptake of ^123^I-MIBG in NE tumours. In nude mice, bearing the human transplantable midgut carcinoid GOT1, all organs and xenografted tumours accumulated ^123^I after i.v. injection of ^123^I-MIBG. A high concentration of ^123^I was maintained in GOT1 tumours and adrenals, which expressed VMATs, but rapidly decreased in all other tissues. In the VMAT-expressing NE tumour cell lines GOT1 and BON and in VMAT-expressing primary NE tumour cell cultures (carcinoids, *n*=4 and pheochromocytomas, *n*=4), reserpine significantly reduced the uptake of ^123^I-MIBG. The membrane pump inhibitor clomipramine had no effect on the uptake of ^123^I-MIBG in GOT1 and BON cells, but inhibited the uptake in one out of four primary carcinoid cell cultures and three out of four primary pheochromocytoma cell cultures. In conclusion, VMATs and secretory granules are of importance for the uptake and retention of ^123^I-MIBG in NE tumours. Information about the type and degree of expression of VMATs in NE tumours may be helpful in future to select patients suitable for radiation therapy with radio-iodinated MIBG.

Amine-producing neurons and neuroendocrine (NE) cells have the capacity to synthesise, store and release amines. Amines are synthesised in the cytoplasm and transported into storage vesicles by specific membrane-bound transporters, that is, vesicular monoamine transporters (VMATs). Once released from the cell, amines can be subject to reuptake by another set of transporters localised to the plasma membrane (Rothman and Orci, 1992; Calakos and Scheller, 1994). Two subtypes of vesicular monoamine transporters, VMAT1 and 2, have been characterised with structural similarities, but with differences in substrate specificity and tissue distribution (Erickson *et al*, 1992; Liu *et al*, 1992; Erickson and Eiden, 1993). In a recent study, we demonstrated the differential expression of VMAT1 and 2 in gut NE tumours, reflecting specific amine production and different cellular origin of these tumours. Serotonin-producing enterochromaffin (EC) cell carcinoids (midgut carcinoids) expressed VMAT1 and 2, while histamine-producing enterochromaffin-like (ECL) cell carcinoids (foregut carcinoids) expressed VMAT2 almost exclusively. Rectal carcinoids and pancreatic endocrine tumours with predominant peptide production expressed VMATs only rarely (Jakobsen *et al*, 2001).

Neuroendocrine tumours can be visualised by scintigraphy after injection of the radio-iodinated noradrenaline analogue meta-iodobenzylguanidine (MIBG), which is a substrate for the amine transporters of chromaffin granules (Henry *et al*, 1994). In clinical studies, the uptake of radiolabelled MIBG was high in pheochromocytomas (derived from the adrenal medulla) in comparison with midgut carcinoid tumours (Kimmig, 1994). Pheochromocytomas express high levels of both VMAT1 and 2, while serotonin-producing midgut carcinoids express mainly VMAT1 (Jakobsen *et al*, 2001). The importance of VMAT expression, functional status or the presence of isoforms of VMAT in NE tumours has not been established.

The aim of the present study was therefore to study the uptake of ^123^I-MIBG in NE tumours in relation to the expression of VMAT isoforms.

## MATERIALS AND METHODS

### Animal model

Male BALB/cABom-nu mice, 3–4 weeks of age (Bomholtgaard, Denmark), were used for transplantation. Tumour tissue was harvested from nude mice, carrying xenografts of the human transplantable midgut carcinoid tumour GOT1, and propagated to new animals every 3 months (Kölby *et al*, 2001). Tumours were minced and small pieces (2 × 2 × 1 mm each) were transplanted to the subcutis in the back of the neck. After 3–4 months, the tumours measuring 10 mm in diameter were used for MIBG uptake studies. The animal experiments were approved by the Ethical Committee for Animal Experiments, Göteborg University.

### Biodistribution and scintigraphy of ^123^I-MIBG in nude mice

The biodistribution of ^123^I-MIBG (Mallinckrodt, Petten, Holland) was studied in eight nude mice carrying GOT1 tumours at 4 h (*n*=2), 24 h (*n*=3) and 48 h (*n*=3) after i.v. injection of 10–12 MBq into the tail vein. Prior to and after the administration of the ^123^I-MIBG, the ^123^I activity in the syringes was measured with a well-type ionisation chamber (CRC-120, Capintec, USA). Tumours and tissue samples from the salivary gland, lung, kidney, heart, spleen, adrenal, small intestine, muscle and blood were collected and weighed. The ^123^I activity was measured in a Wallac 1480 gamma counter (WIZARD3, Wallac Oy, Finland) and the data were corrected for background and physical decay. The calibration factor between the sensitivity of the ionisation chamber and the gamma counter was determined. The ^123^I activity concentration of the radionuclide was expressed as the fraction of injected activity per unit mass of the tissue (%IA/g), and the tumour-to-blood activity concentration ratio, T/B, was determined (Forssell-Aronsson *et al*, 1995). Before killing, whole-body scintigraphies were performed with a gamma camera (General Electric AC/T, UK) equipped with a pinhole collimator.

### ^123^I-MIBG binding in cell cultures

The binding of ^123^I-MIBG was studied in two human NE tumour cell lines (GOT1, derived from a midgut carcinoid; BON, derived from a pancreatic carcinoid) and one non-NE tumour cell line (Colo205, derived from a colon carcinoma) (Semple *et al*, 1978; Evers *et al*, 1991; Kölby *et al*, 2001). GOT1 cells were grown in RPMI 1640 supplemented with 4% foetal calf serum (FCS). BON cells were grown in DMEM+F-12 with 10% FCS. Colo205 cells were grown in Iscove's medium supplemented with 10% FCS. The binding of ^123^I-MIBG was also studied in eight primary tumour cell cultures established from four patients with metastatic ileal carcinoid tumours (cases 1–4) and four with adrenal pheochromocytomas (cases 5–8). Tumour tissues were obtained from primary tumours, or liver metastases, from patients undergoing surgery. The preparation and characterisation of tumour cell cultures have been described previously (Westberg *et al*, 1997). Tumour cells were seeded onto collagen-coated tissue-culture plates (Nunc, IL, USA) at a density of 10^5^–10^6^ cells per well and incubated in RPMI 1640 medium (Northumbria Biologicals, Cramlington, UK) supplemented with 4% FCS. The experiments were performed on tumour cells after 2 weeks in primary culture. Cultures from all tumours contained a majority of tumour cells (>90%) with a minor population of fibroblasts.

The tumour cells were incubated at 37°C for 4 h with 100 *μ*l RPMI 1640 medium containing approximately 500 Bq ^123^I-MIBG (corresponding to 10 nM of MIBG). For blockade of the MIBG binding at the granule membrane or at the plasma membrane, the medium was supplemented with reserpine (1 *μ*M) or clomipramine (1 *μ*M), 30 min before the addition of MIBG, respectively. After incubation for 4 h with ^123^I-MIBG, the medium was removed and the cells were washed rapidly three times with 1 ml Ca- and Mg-free DPBS. The cells were detached from the wells by gentle flushing with 1 ml DBPS containing 5 *μ*g ml^−1^ H-33342 (bis-Benzimide, Hoechst, H-33342), and transferred to a plastic tube. The amount of cell-bound ^123^I was determined by radioactivity measurements of the tube contents using the gamma counter. Correction was made for background and radioactive decay. For DNA quantification, the cells were transferred to a 96-well plate for the measurement of fluorescence intensity, using excitation and emission wavelengths of 355 and 460 nm, respectively (Spectra Max Gemini, Molecular Devices, USA). A standard curve was made by measurements of serial dilutions of DNA (salmon testes DNA, cat. no. D7656, Sigma Chemical Co, St Louis, MO, USA) in DPBS with 5 *μ*g ml^−1^ H-33342. The binding of ^123^I-MIBG to the cell lines was correlated to the amount of DNA in each well. The binding of ^123^I-MIBG was expressed as the fraction of activity administered per *μ*g DNA (%AA *μ*g^−1^). For the primary cell cultures, the cellular binding was expressed as the fraction of activity administered per well.

### Immunocytochemistry

Tissues were fixed in 4% neutral buffered formalin in PBS at pH 7.4. for 4–48 h, dehydrated and embedded in paraffin wax. The sections were incubated overnight with primary antisera (Table 1

**Table 1:** Primary antibodies used for immunocytochemistry and Western blotting

**Antibody**	**Dilution, immuno-fluorescence**	**Dilution, immuno-peroxidase**	**Dilution, Western blotting**	**Clone**	**Cat. no**	**Company**
VMAT1 (goat)	1 : 100	1 : 1000	1 : 2000		sc-7718	Santa Cruz Biotechnology, Inc., Santa Cruz, CA, USA
VMAT1 (rabbit)	1 : 100	1 : 10 000	—			Gift from Jeffrey D Erickson, NIH, MD, USA
VMAT2 (rabbit)	1 : 100	1 : 10 000	—			Jeffrey D Ericksson
VMAT2 (rabbit)	1 : 100	1 : 1000	1 : 2000		AB1767	Chemicon, Temecula, CA, USA
CgA (mouse)	1 : 500	1 : 1000	—	LK2H10	1199021	Boehringer Mannheim, Mannheim, Germany
SV2 (mouse)	1 : 20	—	—			Development Studies, Hybridoma Bank

a —=not tested.

). The bound antibodies were visualised by an indirect immunoperoxidase technique using tyramide signal amplification (TSA-Indirect, NEN Life Science Products, MA, USA). Diaminobenzidine (DAB) was used as a chromogen. The control sections were incubated identically, except for the primary antibodies, which were replaced by normal mouse IgG, normal rabbit or goat serum.

Cells, grown on collagen-coated slides (Chamber Slide®; Nunc Inc.), were fixed in 4% buffered formalin in PBS at pH 7.4 for 4 h, and incubated for indirect immunofluoresence. Preincubation with 5% nonfat dry milk was followed by overnight incubation with primary antisera (Table 1). The bound antibodies were visualised using biotin-labelled secondary antibodies and streptavidin–FITC. Control cultures were incubated identically, except for the primary antibodies, which were replaced by normal mouse IgG or normal rabbit or goat serum. Cultures were examined with a Nikon Eclipse E800 microscope equipped with epi-illumination.

### Western blotting

Tumour biopsies and cultured cells were homogenised in 10 mM potassium-phosphate buffer (pH 6.8). containing 1 mM EDTA, 10 mM CHAPS, 1 *μ*g ml^−1^ aprotinin, 10 *μ*g ml^−1^ leupeptin, 10 *μ*g ml^−1^ pepstatin and 1 *μ*g ml^−1^ Pefablock® (all chemicals from Roche Diagnostics GmbH, Mannheim, Germany). Aliquots of proteins (20 *μ*g) were separated by electrophoresis in precast polyacrylamide gels (10% NuPAGE Bis-Tris-gels; Invitrogen, Carlsbad, CA, USA) using NuPAGE MOPS SDS as a running buffer. Proteins were transferred on to polyvinyldifluoride (PVDF) membranes using a NOVEX blotting system. The membranes were incubated with anti-VMAT1 or anti-VMAT2 antisera followed by alkaline phosphate-conjugated goat anti-rabbit antibodies, and chemoluminescence detection (Table 1). Molecular weight markers (Sea-Blue, Invitrogen) were used to calculate the apparent size of immunoreactive proteins.

### Statistical analysis

For statistical analysis, the Student's *t*-test was used. *P*-values <0.05 were considered to be significant.

## RESULTS

### Biodistribution of ^123^I-MIBG in nude mice with GOT1 xenografts

**Figure 1:**
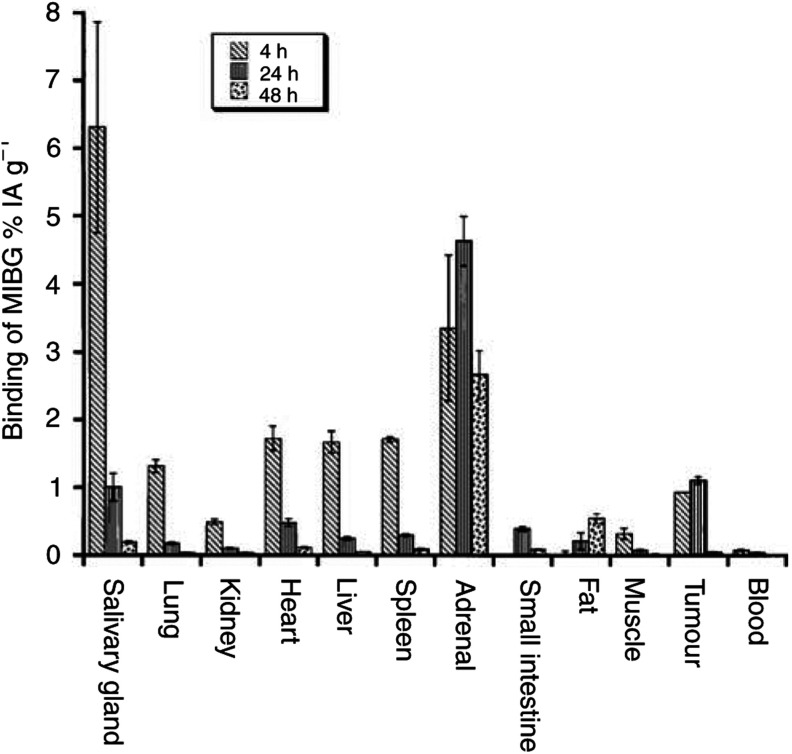
Biodistribution of ^123^I-MIBG in nude mice. Results are given as percent of injected activity per gram of tissue (%IA g^−1^) (mean±s.e.m.). In the tumour and the adrenals, the concentration remained relatively stable over the 48 h period, while the concentration declined rapidly in all other organs.

The activity concentration of ^123^I in tumour tissue was 0. 93±0.06%IA g^−1^ (mean±s.e.m.) IA g^−1^ (*n*=2) at 4 h, 1.1±0.13%IA g^−1^ (*n*=3) at 24 h and 0.54±0.08%IA g^−1^ (*n*=3) at 48 h after injection. The corresponding T/B values were 12±2, 28±5 and 83±13. At 4 h after injection, the concentration in the adrenal was 3.4%IA g^−1^, in the salivary gland 6.3%IA g^−1^ and in the lung, kidney, heart, liver, spleen and muscle it ranged between 0.32 and 1.7%IA g^−1^. In the tumour and the adrenal, the concentration remained stable over the 48 h period, while the concentration declined rapidly in all the other organs (Figure 1

).

**Figure 2:**
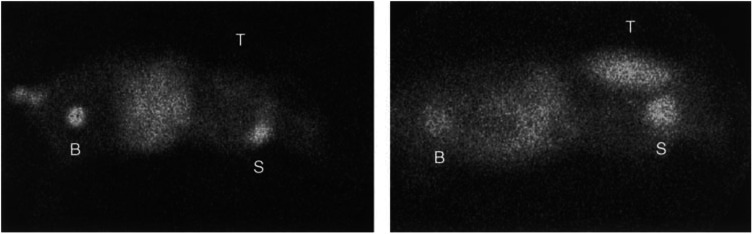
^123^I-MIBG-scintigraphy of nude mice with the GOT1 tumour growing in the back of the neck. At 4 h (left), there was a widespread distribution of ^123^I-MIBG and the tumour was not detectable. After 24 h (right), the uptake in the tumour (T) remained high and it was now clearly visible. Both at 4 and 24 h, the salivary glands (S) and urinary bladder (B) were visible.

Scintigraphy at 4 h showed a widespread distribution of the radionuclide and the tumour was therefore not scintigraphically detectable. At 24 h, the tumour was clearly visible and the radionuclide could also be detected in the salivary glands and urinary bladder. At 48 h, the tumour was no longer scintigraphically detectable (Figure 2

).

### ^123^I-MIBG binding to cultured tumour cells; effects of reserpine and clomipramine

**Figure 3:**
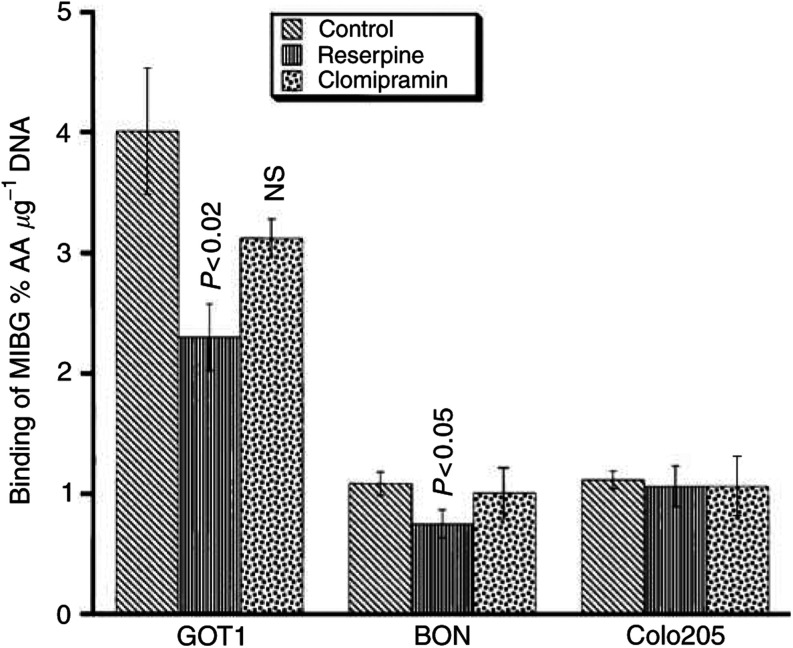
Effect of reserpine and clomipramine on^123^I-MIBG binding to cultured cell lines. Results are given as the percentage (mean±s.e.m.) of administered activity per microgram DNA (%AA *μ*g^−1^). The uptake in GOT1, BON and Colo205 cells was 4.0, 1.1 and 1.1%AA *μ*g^−1^ DNA, respectively. The VMAT antagonist reserpine significantly inhibited the binding in the NE tumour cell lines GOT1 and BON, whereas it had no effect on the non-NE tumour cell line Colo205. Clomipramine had no significant effect on the binding of ^123^I-MIBG in any of the cell lines investigated.

All tumour cell lines investigated accumulated ^123^I-MIBG. Binding to GOT1, BON and Colo205 cells was 4.0, 1.1 and 1.1%AA *μ*g^−1^ DNA, respectively. Reserpine significantly inhibited the binding in the NE cell lines; GOT1: 43% reduction (*P*<0.02) and BON: 31% reduction (*P*<0.05). In the non-NE tumour cell line Colo205, reserpine had no effect on the binding of ^123^I-MIBG. Clomipramine had no significant effect on the binding of ^123^I-MIBG in any of the human cell lines investigated (Figure 3

).

**Figure 4:**
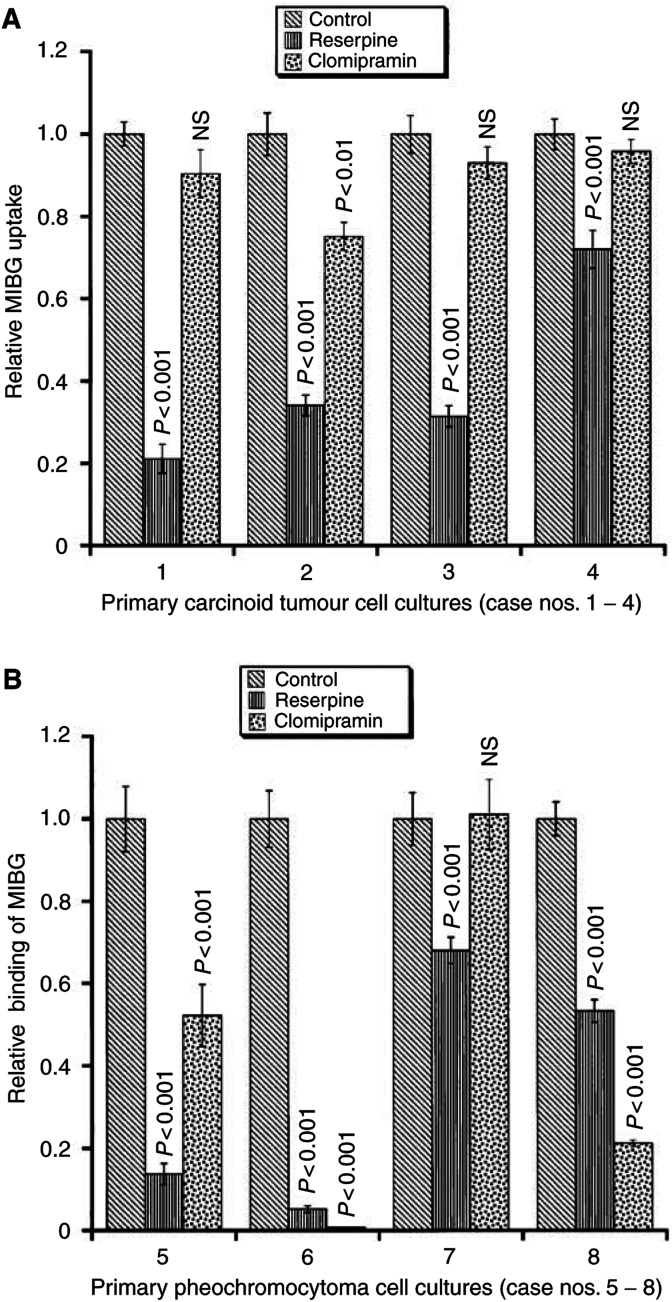
(**A**) Effect of reserpine and clomipramine on^123^I-MIBG binding to primary carcinoid tumour cell cultures. Reserpine strongly reduced the ^123^I-MIBG uptake in all cases. Clomipramine had no effect on ^123^I-MIBG uptake in three out of four ileal carcinoids (case nos. 1, 3 and 4). In contrast, clomipramine had an inhibitory effect on ^123^I-MIBG uptake in one ileal carcinoid (case no. 2). Values are given as mean±s.e.m. (**B**) Effect of reserpine and clomipramine on ^123^I-MIBG binding to primary cell cultures of pheochromocytomas. Reserpine strongly reduced the ^123^I-MIBG binding to all the pheochromocytomas investigated. Clomipramine also reduced the binding to three out of four pheochromocytomas (case nos. 5, 6 and 8), whereas it had no effect on the binding in case no. 7. Values are given as mean±s.e.m.

In the primary cell cultures derived from midgut carcinoid tumours, the binding of ^123^I-MIBG ranged between 1.2 and 21% per well. Reserpine strongly reduced the ^123^I-MIBG binding in the ileal carcinoids; case no. 1: 79% reduction (*P*<0.001); case no. 2: 66% reduction, (*P*<0.001); case no. 3: 68% reduction (*P*<0.001); and case no. 4: 28% reduction (*P*<0.001) (Figure 4a

).

Clomipramine inhibited the binding of ^123^I-MIBG in one ileal carcinoid; case no. 2: 25% reduction (*P*<0.01). In contrast, clomipramine had no effect on ^123^I-MIBG binding in the other ileal carcinoids (case nos. 1, 3 and 4) (Figure 4A).

In primary cell cultures derived from pheochromocytomas, the binding of ^123^I-MIBG ranged between 0.94 and 15% per well. Reserpine strongly reduced the ^123^I-MIBG binding in all pheochromocytomas investigated; case no. 5: 86% reduction (*P*<0.001); case no. 6: 95% reduction (*P*<0.001); case no. 7: 32% reduction (*P*<0.001); and case no. 8: 47% reduction (*P*<0.001).

Clomipramine also reduced the binding in three of four pheochromocytomas; case no. 5: 48% reduction (*P*<0.001); case no. 6: 99% reduction (*P*<0.001); and case no. 8: 79% reduction (*P*<0.001). In contrast, clomipramine had no effect on ^123^I-MIBG binding in case no. 7 (Figure 4B).

### Expression of VMAT1 and 2, SV2 and CgA

Immunocytochemical analysis showed that xenografted GOT1 tumours and the adrenal medulla of host animals were strongly positive for VMAT1 and 2. The VMAT1 and 2 immunoreactive material was located in the secretory granules in the cytoplasm of tumour cells and adrenal chromaffin cells. All the other organs investigated were negative for VMAT1 and 2. The NE tumour cell lines GOT1 and BON were both positive for VMAT1 and 2, SV2 and CgA immunocytochemically. The immunoreactive material was located in secretory granules in the cytoplasm of the tumour cells. Colo205 tumour cells were negative for all these markers (Table 2

**Table 2:** Expression of VMAT1 and 2 and secretory granule markers in cell lines analysed by immunocytochemistry

	**Immunocytochemistry**			
	**VMAT1**	**VMAT2**	**SV2**	**CgA**
GOT1	+	+	+	+
BON	+	+	+	+
Colo205	−	−	−	−

+=present; −=absent.

and Figure 5

**Figure 5:**
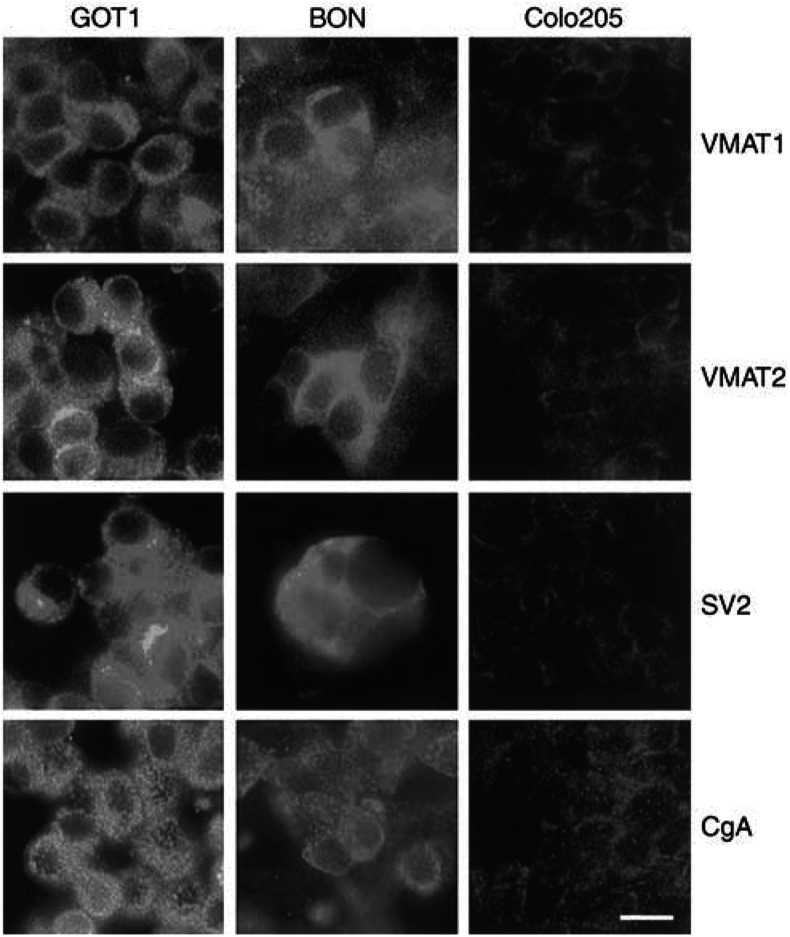
Immunocytochemical examination of the NE tumour cell lines GOT1 and BON and the non-NE tumour cell line Colo205. GOT1 and BON cells were both positive for VMAT1 and 2, SV2 and CgA. Colo205 cells were negative for all these markers. The scale bar represents 10 *μ*m.

). Immunofluorescence analysis of primary cell cultures demonstrated VMAT1 in four of four carcinoids and three of four pheochromocytomas, whereas VMAT2 was present in three of four carcinoids and four of four pheochromocytomas. Immunoperoxidase staining of tumour biopsies demonstrated the expression of VMAT1 in seven of eight tumours and VMAT2 in eight of eight tumours (Table 3

**Table 3:** Expression of VMAT1 and 2 in primary cell cultures (MC=midgut carcinoid, PC=pheochromocytoma)

	**Immunocytochemistry**					
	**Biopsy −/+−+++ scale^a^**		**Cell culture −/+ scale**		**Western blot −/+ scale**	
**Primary cell culture no./tumour type**	**VMAT1**	**VMAT2**	**VMAT1**	**VMAT2**	**VMAT1**	**VMAT2**
1 MC	+++	++	+	−	+	−
2 MC	+++	+++	+	+	+	+
3 MC	+++	+++	+	+	+	+
4 MC	+++	+++	+	+	+	+
5 PC	+++	+++	+	+	+	+
6 PC	+++	+++	+	+	+	+
7 PC	−	+++	−	+	−	+
8 PC	+++	+++	+	+	+	+

+=1–25% of tumour cells positive; ++=25–75% of tumour cells positive; +++=75–100% of tumour cells positive.

).

Western blotting of tumour biopsies confirmed the expression pattern obtained by immunocytochemical analysis (Table 3). VMAT1 migrated as one broad band with a molecular weight of 65–95 kDa, while VMAT2 appeared in two bands: a major band at 65–90 kDa and a minor band at 40–50 kDa.

## DISCUSSION

Radio-iodinated MIBG can be used for scintigraphic visualisation of NE tumours by scintigraphy and for targeted radiation therapy. Pheochromocytomas and neuroblastomas rapidly accumulate MIBG and are therefore efficiently visualised by MIBG scintigraphy, while only about one-third of carcinoid tumours can be visualised by this technique (Kimmig, 1994). The reason why certain carcinoid tumours cannot be visualised has not been explained, and the precise mechanism behind MIBG uptake and retention in NE tumours is unclear. It has been shown that MIBG is a substrate for VMATs, located on the membrane of secretory vesicles of NE cells (Henry *et al*, 1994; Erickson *et al*, 1996). It is therefore likely that after passing the plasma membrane, MIBG is transported into secretory granules by VMATs. In this study, we have demonstrated that ^123^I was widely distributed to several organs of nude mice with GOT1 xenografts 4 h after the administration of ^123^I-MIBG. However, the long-term retention of radioactivity was only observed in tissues expressing high levels of VMATs, viz. tumour xenografts and adrenals. This finding was corroborated by the results from the NE and non-NE tumour cell lines. All the cell lines showed binding of ^123^I-MIBG, but only NE cell lines expressing VMATs had their binding reduced by the VMAT antagonist reserpine. These results indicate that the secretory granules containing VMATs are of importance for the uptake and retention of MIBG in tumour tissues.

The specific role of plasma membrane transporters for the binding of MIBG in tumour cells still remains unclear. Previous studies on midgut carcinoid tumours in primary culture have indicated the presence of transporters at the plasma membrane in these tumours (Wängberg *et al*, 1990). However, inhibition of the membrane pump by clomipramine had no effect on MIBG uptake in any of the carcinoid tumour cell lines investigated. The results from nude mice with widespread distribution of MIBG in several organs, together with the negative results with clomipramine in the cell line studies, indicate alternative transport mechanisms for MIBG to pass the plasma membrane of carcinoid tumour cells. This was corroborated by the results from primary carcinoid cell cultures, which in three out of four cases, did not respond to clomipramine. On the other hand, clomipramine markedly reduced the uptake of MIBG in almost all catecholamine-handling primary pheochromocytoma cell cultures. This indicates that pheochromocytomas predominantly use the noradrenaline transporter for the uptake of MIBG across the plasma membrane.

Immunocytochemical analysis of tumour biopsies revealed that all tumours examined expressed VMAT1 and/or VMAT2. The quantitative expression of each VMAT *vs* MIBG uptake and retention was not determined in the present experiments. The expression of VMAT was maintained in the cell culture. The specific uptake of MIBG in cultured tumour cells was demonstrated in VMAT-expressing tumours (both carcinoids and pheochromocytomas) and this uptake was antagonised reserpine. In primary cell culture, blockade of the noradrenergic membrane pump by clomipramine was effective only in one out of four carcinoids and in three out of four pheochromocytomas. These results confirm the hypothesis that VMATs are of importance for the uptake of MIBG in amine-handling NE tumour cells.

In conclusion, this study shows that VMATs are of primary importance for uptake and retention of the catecholamine analogue MIBG in NE tumour cells. The storage of MIBG in secretory granules was markedly reduced by the VMAT antagonist reserpine. A common mechanism behind MIBG transport across the plasma membrane could not be shown in these experiments. Pheochromocytoma cells utilised the noradrenaline transporter, antagonized by clomipramine. On the other hand, uptake of MIBG into carcinoid cells was antagonised by reserpine, but only occasionally by clomipramine. A better understanding of the mechanisms behind MIBG uptake in NE tumours will be helpful to select patients suitable for therapy with radio-iodinated MIBG. Drug-promoting uptake and retention of MIBG will be of interest to enhance the therapeutic effects.
